# Crosstalk between the Resident Microbiota and the Immune Cells Regulates Female Genital Tract Health

**DOI:** 10.3390/life13071531

**Published:** 2023-07-09

**Authors:** Luigi Santacroce, Raffaele Palmirotta, Lucrezia Bottalico, Ioannis Alexandros Charitos, Marica Colella, Skender Topi, Emilio Jirillo

**Affiliations:** 1 Microbiology and Virology Section, Interdisciplinary Department of Medicine, School of Medicine, University of Bari “Aldo Moro”, 70124 Bari, Italy; raffaele.palmirotta@uniba.it (R.P.); emilio.jirillo@uniba.it (E.J.); 2 Department of Clinical Disciplines, School of Technical Medical Sciences, “Alexander Xhuvani” University of Elbasan, 3001 Elbasan, Albania; 3 Respiratory Rehabilitation Unit, Clinical Scientific Institutes Maugeri (IRCCS), 70124 Bari, Italy

**Keywords:** female genital tract, microbiota, immunity, lactoferrin, polyphenols, probiotics

## Abstract

The female genital tract (FGT) performs several functions related to reproduction, but due to its direct exposure to the external environment, it may suffer microbial infections. Both the upper (uterus and cervix) and lower (vagina) FGT are covered by an epithelium, and contain immune cells (macrophages, dendritic cells, T and B lymphocytes) that afford a robust protection to the host. Its upper and the lower part differ in terms of *Lactobacillus* spp., which are dominant in the vagina. An alteration of the physiological equilibrium between the local microbiota and immune cells leads to a condition of dysbiosis which, in turn, may account for the outcome of FGT infection. Aerobic vaginitis, bacterial vaginosis, and *Chlamydia trachomatis* are the most frequent infections, and can lead to severe complications in reproduction and pregnancy. The use of natural products, such as probiotics, polyphenols, and lactoferrin in the course of FGT infections is an issue of current investigation. In spite of positive results, more research is needed to define the most appropriate administration, according to the type of patient.

## 1. Introduction

The female genital tract (FGT) consists of the uterine cervix, fallopian tubes, vagina and vulva, which perform various functional steps, such as the passage of the spermatozoa during insemination, and the expulsion of the fetus and placenta during delivery, and of the exfoliated endometrium during the menstrual period [1,2].

Because of its direct exposure to the external environment, the FGT is very susceptible to suffering microbial infections and inflammation. Under steady-state conditions, protection from invading pathogens is afforded by epithelial cells, resident immune cells, and the microbiota [3]. The cervix and vagina are covered by a stratified squamous epithelium, which represents a barrier against pathogens, as demonstrated by the evidence that its destruction increases the risk of HIV infection [4]. Innate immunity is based on the function of uterine natural killer (NK) cells, which recognize and lyse infected cells [5].

Macrophages represent the second most copious contingent of immune cells in the endometrium, which engulf and kill microbes, as well as self-cells [6].

As far as adaptive immunity is concerned, it is mostly T cells that are found in cervical and vaginal tissues, with tissue-resident memory (TREM) cells protecting against pathogens [7].

The cervico-vaginal microbiota constitute a complex micro-ecosystem, which play a critical role in maintaining homeostasis. The cervico-vaginal microbiota and their main functions have been well known for a long time, thanks to Albert Döderlein’s 1892 work that discovered and described the main features of certain vaginal acidogenic bacteria. However, the uterine microbiota have been less investigated than those of the vagina. Their composition appears to depend on disease status, such as in the case of endometrial cancer, and implantation failure [8,9].

*Lactobacillus* spp. form the predominant genus in the healthy FGT, providing several beneficial effects to the host. In fact, they maintain the vaginal pH at 3.8–4.5, through the production of lactic acid by the vaginal epithelial cells. In turn, the lactic acid regulates inflammation in the acidic microenvironment, while inhibiting the proliferation of other microorganisms, and competing with pathogens for survival and nutrition.

According to a few reports, *Lactobacillus* spp. Are not often detected in the endometrium, where a prevalence of *Gardnerella (G.) vaginalis*, *Enterobacter*, and *Streptococcus (S.) agalactiae* has been observed [10,11,12,13,14,15].

In the vagina, the microbiota are influenced by hygienic conditions, temporal dynamics, menstrual cycles, hormone levels, and concurrent diseases [16,17].

An alteration of the FGT microbiota may lead to a condition of so-called dysbiosis, with an increase in pathogenic bacteria over *Lactobacillus* [18,19,20]. In this respect, the main dysbiosis-mediated infections include vaginitis, endometritis, cervicitis, and pelvic inflammatory disease (PID) [21,22,23].

Aerobic vaginitis (AV), bacterial vaginosis (BV), and *Chlamydia trachomatis (C. t.)* infections represent the most-studied FGT infections.

In women of childbearing age, the incidence of AV is about 10%, with a prevalence of aerobic bacteria or *Enterococcus* [24]. BV is the most frequent vaginal disease in childbearing-age women, with serious consequences, such as infertility, miscarriage, the premature rupture of membranes, premature delivery, and an increased risk of sexually transmitted infections [25,26]. Microbiologically, BV is characterized by a decrease in *Lactobacillus* and an increase in anaerobic and microaerobic bacteria [27]. The prevalence of *C. t.* in women aged 15–49 years is 1.5–7%, with an asymptomatic clinical course. As lactic acid is an inhibitor of *C. t.* growth, vaginal microbiota containing *Lactobacillus iners*, which produces less lactic acid, promote the risk of *C. t.* infection [28,29]. Among 10% of *C. t.*-infected women, a progression toward PID has been reported, with the risk of severe ectopic pregnancy, abnormal reproductive function, and cancer [30].

Novel therapeutic approaches to improve the function of the FGT immunity–microbiota axis are mainly represented by the administration of natural compounds; i.e., probiotics, lactoferrin (LF), and polyphenols.

Probiotics exert several beneficial effects, even including the release of antimicrobial substances, and the modulation of the immune system [31,32,33]. LF is an antimicrobial peptide (AMP) that is able to prevent bacterial and fungal infections in the FGT [34]. Polyphenols are endowed with antioxidant, anti-inflammatory, and microbicidal activities [35], but data related to their effects on FGT infections are still experimental.

In the present review, an emphasis will be placed on the description of the resident immune cells and the microbiota of the FGT. Then, the main dysbiosis-mediated FGT infections will be elucidated. Finally, novel therapeutic attempts to treat FGT infections with natural products will be discussed.

## 2. Anatomy of the Female Genital Tract

The FGT consists of the lower FGT (vagina and ectocervix), with a large colony of microbes, and the upper FGT (endocervix, uterus, and oviduct) which, conversely, is sterile. Histologically, the cervix is composed of stroma and epithelial cells with an extracellular matrix, including type I and type II collagen. Type I collagen, which represents 70% of the extracellular matrix, maintains tissue integrity. The endocervix is covered by a columnar epithelium, while the ectocervix and vagina are covered by continuous stratified non-keratinized squamous epithelial cells. The area between the endocervix and the ectocervix is the so-called transformation zone, which is composed of squamous columnar epithelial cells. Columnar epithelial cells are composed of tight junctions that are regulated by estrogen, cytokines, and growth factors. On the other hand, the squamous epithelium contains adhesive junctions and desmosome junctions, which permit the passage of small molecules through the intercellular space. Notably, epithelial cells can act as presenting cells, thus recognizing microbial antigens and secreting antibiotics, cytokines, and chemokines.

## 3. Main Features of the Cervical Mucus

Cervical mucus is a product of goblet/secretory cells, in the context of the crypts. Mucins are the main constituents of cervical mucus, with mainly MUC5B and MUC5AC contained in the cervix. MUC5B reaches its highest level at the ovulation phase, with a watery secretion, which permits the entry of sperm into the upper reproductive tract. Conversely, during the luteal phase, the MUC5B expression level is decreased by progesterone, thus leading to a thickening in the cervical mucus. There is evidence that the menstrual cycle determines a bimodal distribution of the cervical immune components. Taking into consideration IgG/IgA, lactoferrin, interleukin (IL)-10, and antimicrobial peptides, these factors are expressed at higher levels at the early stage of the follicular phase, followed by a dramatic decrease in the middle of menstruation, and a further increase at the latter stage of menstruation.

In general terms, the cervical mucus represents a defense barrier, with mucin able to capture pathogens; meanwhile, the local immune response begins to act. In this last respect, lactoferrin, IgG, and IgA inhibit the adhesion and invasion of the cervical cells by pathogenic microorganisms [36].

## 4. The Immune Arsenal of the FGT

### 4.1. Innate Immunity

The epithelial cells of the FGT are non-immune cells that mostly defend the lower reproductive tract, cervix, and vagina. A stratified squamous epithelium, where tight junctions (TJ) predominate, covers the lower tract, while the upper portion, fallopian tubes, uterus, and inner cervix are characterized by a monolayer columnar epithelium, endowed with a more tightly connected network [37,38]. The effectiveness of the epithelial barrier of the FGT is supported by the evidence that its destruction appears to promote HIV infection, as well as the recruitment of HIV target cells. As was recently reported, the treatment of bovine endometrial epithelial cell lines with astaxanthin, a carotenoid, could reduce the lipopolysaccharide (LPS)-induced release of pro-inflammatory cytokines, while increasing the activity of cell superoxide dismutase and catalase, insulin-like growth factor, and epithelial growth factor [39]. In particular, such a treatment led to an increased expression of claudin, a TJ involved in the maintenance of the epithelial defense barrier against pathogen invasion.

Thanks to the toll-like receptors (TLRs) expressed on their membrane, epithelial cells from FGT are able to bind specific molecules of gram-positive and gram-negative bacteria, e.g., peptidoglycans and LPS, respectively [40], thus secreting anti-microbial peptides (AMPs) and cytokines [41,42,43,44]. AMPs, such as human beta defensin (HBD), LL-37, S200 protein, lysozyme, and iron metabolism proteins, prevent invasion by microbes in the upper FGT, while regulating the vaginal microbiota [45].

Moreover, epithelial cells from the FGT release a plethora of cytokines, i.e., interleukin (IL)-1 alpha, IL-1 beta, and tumor necrosis factor (TNF)-alpha, in response to *Prevotella*, *Mobiluncus*, and *Sneathia*, in the course of BV [46,47].

Natural killer (NK) cells are mostly confined to the uterus and perform a protective function against pathogens [48]. Functionally, NK cells protect the placenta from *Listeria* infection, and prevent the infection-induced abortion via secretion of granulysin into the placental trophoblast [49].

Macrophages are very abundant in the endometrium, playing a double role in the context of the FGT [50]. In fact, they engulf and kill pathogens, thus contributing to local innate immune defenses [51]. On the other hand, they maintain a tolerance milieu in the FGT, via the release of interleukin (IL)-10, transforming growth factor (TGF)-beta, and indoleamine (IDO) [52,53].

Dendritic cells (DCs) are the major APCs of the immune system and, via the activation of T regulatory (TREG) cells, maintain a steady-state condition in the FGT microenvironment [54]. Conversely, evidence has been provided that in the context of the cervical mucosa, DCs can capture and transfer HIV-1 via Siglec-1, thus facilitating viral dissemination [55].

### 4.2. Adaptive Immunity

In the context of the FGT, T and B lymphocytes represent the major players. In the human cervical mucosa and vagina, antigen-specific TRM CD8+ cells have been identified, and T-cell-induced vaccines are able to prevent FGT HIV infection [56,57]. Quite interestingly, estradiol has been shown to increase the TRM CD4+ cell response to the *Herpes simplex* (HSV) virus, via IL-17 secretion [58]. With special reference to the contingent of FGT Treg cells, they maintain the local immune tolerance, also attenuating the inflammatory response in the case of microbial stimulation of the immune system [59].

B cells are scarcely present in the FGT mucosa and, just recently, it has been reported that migrant memory B cells secrete specific IgG-2b anti-HIV in the vagina [60].

**Figure 1:**
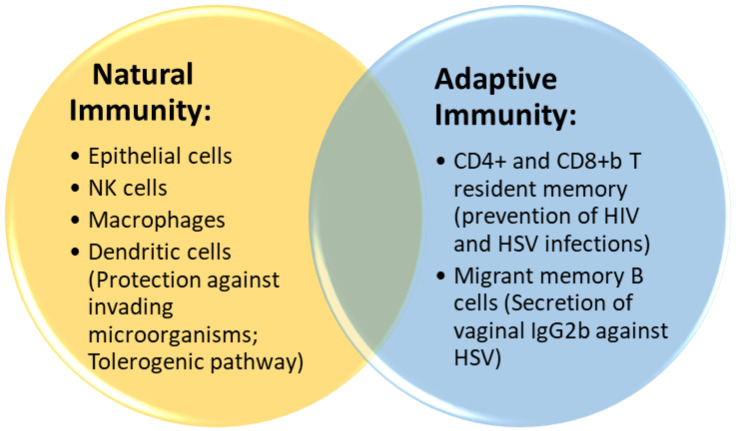
Local immunity in the FGT. Natural immunity and adaptive immunity contribute to the protection of the mucosal FGT, and also to maintaining a tolerogenic profile of the genital habitat.

The immune components of the FGT are described in Figure 1.

Studies on the effects of the menstrual cycle and menopause on cervical T lymphocytes are controversial. For instance, the total number of CD8 + T cells and CD4 + T cells in the cervix remains unaltered, even including CD8 + T cell toxicity. Notably, transforming growth factor (TGF)-beta plays a dual role, inhibiting endometrial CD8 + T cells in the secretory phase, while TGF-beta does not act on cervical CD8 + T cells. This still allows a protective role by the cervix against pathogens, when the secretory endometrium is immunosuppressed by embryo implantation. Conversely, other studies report that the menstrual cycle and age impair the immune function. The levels of chemokine (C-C) motif ligand 2 (CCL2) and local CD4+ TRM cells increase during the follicular phase, because the inhibitory effect of progesterone decreases, and more CD8+ TRM cells are recruited to the cervix.

Furthermore, the frequency of CD8 + T cells and DCs decreases with age in the cervical tissue of postmenopausal women, thus supporting the increased susceptibility of these women to genital infections.

## 5. Composition and Function of the FGT Microbiota

With special reference to microbial communities, there are differences between the upper and lower tract of the FGT, with a higher bacterial load in the latter [61,62]. Mostly, the vaginal microbiota have been the object of intensive investigation, which has led to the identification of five separate community state types (CSTs) [63,64,65]. *Lactobacillus* represents the major microbiota of the vagina, with *Lactobacillus (L.) crispatus*, *L. gasseri*, *L. iners*, and *L. jensenii* very dominant [66]. Notably, the microbiota that are non-*Lactobacillus* prevalent appear to be associated with disease status and inflammation [67].

Among CSTs, CST-I, CST-II, and CST-V, which contain higher levels of *L. crispatus*, *L. gasseri*, *L. iners*, and *L. jensenii*, are characterized by a low pH, a high concentration of lactic acid, and a less inflammatory status [68,69,70]. Conversely, CST-IV, dominated by bacteria such as *Atopobium*, *G. vaginalis*, *Mobiluncus*, *Prevotella*, *Anaerococcus*, and *Sneathia*, are somewhat associated with BV [71,72].

The composition of the vaginal microbiota seems to influence the epithelial barrier function, as in the case of *L. crispatus*, which is associated with mucus that is able to trap HIV more effectively than other types of *Lactobacillus* spp. do [67]. On the other hand, BV-associated bacteria produce sialidase, which degrades sialic acid, a critical component in the mucus [68].

The vaginal levels of AMPs are associated with vaginal microbial diversity, and higher amounts of HBD-2, lactoferrin (LF), and LL-37 have been detected in the cervico-vaginal lavage of women with BV [73,74,75].

It is worth mentioning the role played by microbial metabolites in the vagina. The levels of D-lactic acid (D-LA) and L-lactic acid (L-LA) produced by the vaginal *Lactobacillus* greatly contribute to vaginal health. In fact, both reduce the pH of the vaginal mucosa, preventing the growth of pathogens, and also reducing the release of pro-inflammatory cytokines by epithelial cells [76]. In particular, LA in vitro induced the release of the anti-inflammatory cytokine, Il-1 receptor antagonist, and inhibited the TLR-mediated production of pro-inflammatory cytokines, in response to seminal plasma [77]. Furthermore, *Lactobacillus* spp. produce hydrogen peroxide and bacteriocins, which inhibit the growth of pathogens [78,79]. Furthermore, cervicovaginal supernatants of *L crispatus* could restore the endocervical barrier integrity, otherwise impaired by *G. vaginalis* culture supernatants [80].

A few studies have been focused on the role of vaginal short-chain fatty acids (SCFAs), for their protective role at the gut-mucosa level [81]. There is evidence that SCFAs are elevated in the vaginal mucosa of BV patients [82].

According to another report, high levels of SCFAs could, in vitro, trigger the release of pro-inflammatory cytokines from vaginal and cervical epithelial cells [83]. Taken together, the above results suggest that more studies are required, to clarify the exact role of SCFAs in the FGT.

**Figure 2:**
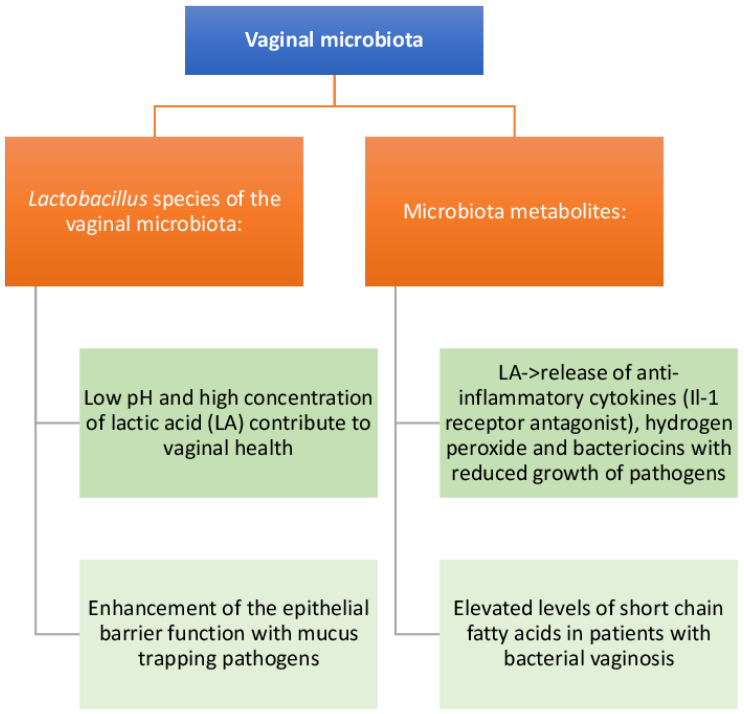
Composition and functions of the vaginal microbiota. *Lactobacillus* spp. and their metabolites are able to increase the protective epithelial barrier, also acting as microbicidal and anti-inflammatory agents. pH < 4.5; *L. a.* concentration 120 mm; SCFAs concentration: acetate <120 mm.

The composition and function of the FGT microbiota are illustrated in Figure 2.

## 6. Dysbiosis and FGT Infections

Both the microbiota and metabolites undergo modifications during cervicovaginal dysbiosis. *Lactobacilli* utilize sugars, such as glycogen and glycogen hydrolysates, to produce lactic acid that, together with bacteriocins and biosurfactants, protect the cervicovaginal milieu. As described in the following paragraphs, BV is one of the most frequent forms of dysbiosis in the vagina, due to a decrease in *Lactobacilli* and an increase in anaerobic bacteria. At the same time, metabolites are also altered during cervicovaginal dysbiosis, with high levels of biogenic amines and short-chain fatty acids, and low levels of some amino acids, such as tyrosine and glutamate. Conversely, the metabolic signature of AV is less defined than that of BV [84,85,86].

In the following paragraphs, the major FGT infections will be described, in relation to the condition of dysbiosis.

### Aerobic Vaginitis

AV patients harbor increased numbers of aerobic bacteria or Enterococcus in the vagina. Among these, *Escherichia (E.) coli*, *S. agalactiae*, *S. anginosus*, *Staphylococcus (S.) aureus*, *S. epidermidis*, and *Enterococcus faecalis* are the main ones.

With special reference to the pathogenic mechanisms elicited by AV-related bacteria, *Staphylococcus* spp. in the vagina cause an increase in SCFAs, especially acetate, as well as the triggering of the anti-inflammatory pathway sustained by Treg cells [87,88,89]. Conversely, in AV, other uropathogens are able to increase the expression of an array of pro-inflammatory cytokines, even including IL-1 beta, IL-6, IL-8, and TNF-alpha, as an indication of the local immune imbalance between inflammatory and anti-inflammatory forces [90,91]. Quite interestingly, some bacteria, in the course of AV, become resistant to the host immune response, as in the case of *S. aureus*, which evade the microbicidal activity locally produced by nitric oxide (NO) [92,93]. Moreover, *S. aureus* upregulates the NO-inducible lactate dehydrogenase, thus surviving to lactic acid fermentation [94]. In the same manner, the toxic shock syndrome toxin-1 (TSST-1) strains elude the local immune response, and induce the release of pro-inflammatory cytokines from vaginal epithelial cells, with a severe damage of the mucosal barrier [95].

There is evidence that, in BV patients, the vaginal microbiota are subverted with a decrease in *Lactobacillus* spp. and an increase in anaerobic bacteria and microprobes, e.g., *G. vaginalis*, *Atopobium*, *Mycoplasma*, *Megasphaera*, *Mobiluncus*, *Roseburia*, *Diadister*, *Sneathia*, and *Prevotella* spp. [96,97]. Notably, in BV patients, the metabolites of the genital tract appear to be more specific indicators of the disease phenotype than the bacteria do [98]. For instance, 2-hydroxyisovalerate and gamma-hydroxybutyrate are increased in the course of BV, with a decrease in lactic acid and tyrosine [99]. Furthermore, the production of succinic acid by *Prevotella* spp. and *Mobiluncus* spp. leads to the inhibition of leukocyte chemotaxis, and the modulation of the immune response. Moreover, the release of SCFAs in combination with bacteria influences the vaginal immune response, either recruiting neutrophils and monocytes, or inhibiting the release of pro-inflammatory cytokines [100].

*C. t.* infection is widely diffused among women aged 15–49 years, and may progress toward severe complications, such as ectopic pregnancy, reproductive abnormalities, and cancer [101]. From a pathogenic point of view, the predominance of *L. iners* in the vagina increases the risk of *C. t.* infection, as it produces less lactic acid [102]. In this respect, the production of *L. iners* for therapeutic use has started [103].

Furthermore, in *C. t.*-infected patients, the vaginal levels of valine, isoleucine, tyramine, cadaverine, and succinate were lower than those detected in healthy people [104]. This may indicate that *C. t.* likely utilizes nitrogen as a nutrient source or affects the nitrogen metabolism of the host. INDO is largely produced by *Prevotella* spp. in the course of BV, and it may promote *C. t.* overgrowth [105,106].

*C. t.* exploits INDO, to activate the synthesis of tryptophan, thus neutralizing the inhibition of *C. t.* growth mediated by interferon (IFN)-gamma [102]. In fact, IFN-gamma promotes the synthesis of indoleamine 2,3-dioxygenase with the consumption of tryptophan required for the growth of *C. t.* [107].

Another mechanism of escape by *C. t.* relies on its property of depriving the inducible nitric oxide synthase (iNOS) substrate, arginin, ultimately abrogating the microbicidal activity of NO [108].

The evasion of the immune response by *C. t.* leads to a condition of chronic infection, where T helper (h)1, Th2, and Th17 lymphocytes become activated, provoking damage, fibrosis, and scarring of the FGT [109,110].

The pathogenic mechanisms of FGT infections are expressed in Table 1.

## 7. Treatment with Natural Products for Maintaining the Health of the FGT

As discussed in the previous sections of this review, the dysbiosis of the FGT leads to different pathologies, even including infectious events. In particular, the misuse and abuse of antibiotics reduces the *Lactobacillus* contingent in the vagina, promoting a resistance to antimicrobials, with the generation of multi-resistant microorganisms [111,112,113,114]. Therefore, attempts have been made to normalize the vaginal microbiota with the supplementation of natural products.

Among these products, probiotics are currently used in FGT infections [115]. By definition, probiotics are “live microorganisms that, when administered in adequate amounts, confer a health benefit to the host” [116].

As was recently reported [117,118], for the treatment of FGT infections, probiotics can be administered in various combinations, i.e., vaginal/oral capsules, vaginal/oral powders, ovules, and sanitary towels. As far as the mechanisms of action of probiotics are concerned, they include the production of antimicrobials (bacteriocins, lantibiotics) and enzymes (arginine deaminase), promotion of adherence to epithelial cells, release of the components of mucus, extracellular matrix-colonization permanence, competition for nutrients, and modulation of the immune system [119,120].

Among relevant clinical trials, evidence has been provided that the administration of four *Lactobacillus* species could increase the vaginal colonization of *Lactobacillus*, followed by an improvement in the clinical symptoms of the infection [121,122,123].

Concerning the local administration of probiotics, vaginal suppositories increased the total number of vaginal *Lactobacillus* bacteria after seven days of treatment, in view of their ability to adhere to, and colonize, the vaginal epithelium [124]. A Lactin-V (L. CTV-05) vaginal treatment gave rise to a lower incidence of BV recurrence [125]. In a series of trials based on the administration of probiotic strains to healthy women, in patients with AV and BV, or in women with vulvo-vaginal candidiasis, an absence of side effects, the colonization and permanence of *Lactobacillus*, and reduced number of recurrences were observed [126,127,128].

Commercial products have been shown to be very effective in the course of FGT infections. Trivagin°, a product enriched in *L. rhamnosus*, *L. gasseri*, *L. fermentum*, and *L. plantarum* was very effective in normalizing the vaginal microbiota and attenuating symptoms of inflammation [129]. In another trial, women with BV who were treated with Ecovag° vaginal gelatin capsules, containing two strains of *Lactobacillus*, demonstrated resolution of disease at the end of the six-month follow-up [130]. Floridia^®^ vaginal tablets, composed of *L. brevis* CD2, *L. salivarius* FV2, and *L. plantarum* FV9, were very effective in the eradication of BV-related bacteria, with a decrease in pro-inflammatory cytokines and reactive oxygen species (ROS) [131,132]. Notably, probiotic suppositories are usually devoid of major side effects, but vaginal discharge has been reported in some studies [113,119,120,133].

Despite the above-cited positive results, further trials are needed, to establish the efficacy of probiotics in pregnant and post-menopausal women, and for the prevention of preterm birth.

Polyphenols are natural substances that are largely contained in fruits, vegetables, cereals, extra virgin olive oil, and red wine [35,134]. They are endowed with antioxidant, anti-inflammatory, and antimicrobial effects and, therefore, are currently used in different pathologies [135,136]. With special reference to FGT infections, a recent paper has demonstrated the anti-inflammatory activity of polydatin, a polyphenol extracted from the rhizome of *Polygonum cuspidatum*, in the course of LPS-induced murine endometritis [137]. Polydatin was able to deactivate the NF-kB pathway, with a dramatic reduction in pro-inflammatory cytokine release. Therefore, it was proposed as a potential remedy in the case of human endometritis. A resveratrol-loaded liposome has been prepared for the topical treatment of vaginal inflammation and infections, in view of its in vitro scavenging activity, and its inhibition of pro-inflammatory cytokine release [138].

Lactoferrin is an AMP endowed with antimicrobial and immunomodulating properties, secreted by the uterine and vaginal epithelial cells [139,140,141]. The LF levels in cervicovaginal fluid increase in the course of FGT infections, correlating with the number of infiltrating neutrophils [142,143]. In a series of clinical trials in women with dysbiosis of the FGT and bacterial and fungal infections, the oral and/or intravaginal administration of LF led to clinical and histological improvements, with a dramatic reduction in pathogens and biomarkers of inflammation [144,145,146,147]. Of note, LF from bovine milk has been used as a dietary supplement, acting as a prebiotic on the intestinal microbiota, and increasing the colonization of *Lactobacillus* strains in the vagina [148].

The effects of natural-product administration on FGT infections are described in Table 2.

## 8. Conclusions

In summary, the network between the FGT microbiota and the local immune system plays a fundamental role in the maintenance of homeostasis, with special reference to the vaginal milieu. Any disturbance of the above indicated equilibrium may lead to a disease status; i.e., AV, BV, and *C. t.* infection, which may culminate in female infertility. The correction of FGT dysbiosis using natural products is a field of current interest, and clinical trials using probiotics and LF have led to positive results. Polyphenols, despite their well-known antioxidant, anti-inflammatory, and antimicrobial activities, have been investigated less.

In general, further data are needed, to define the exact interactions between FGT microbes and natural products, as well as in relation to the disease status of patients.

## Figures and Tables

**Table 1:** Pathogenetic mechanisms responsible for vaginal infections. The bacteria responsible for infection perform different activities, aimed at decreasing the vaginal antimicrobial function, or utilizing substances necessary for these microbes’ growth.

Disease	Pathogenetic Mechanisms
Aerobic vaginitis	Increased production of pro-Inflammatory cytokines Production of oxide-inducible lactate dehydrogenase with survival to lactic acid fermentation (*S. aureus)*
Bacterial vaginosis	Reduced release of lactic acid and tyrosine Increased release of succinic acid with inhibition of leukocyte chemotaxis
*Chlamydia trachomatis* (*C. t.*) infection	Less production of lactic acid Reduced vaginal levels of valine, tyramine, cadaverine and succinate Utilization of tryptophan for its growth Abrogation of anti-microbial activity of nitric oxide

**Table 2:** Main natural products used in the correction of FGT dysbiosis. Probiotics, lactoferrin, and polyphenols attempt to reconstitute the normal microbiota, and also conduct anti-inflammatory, antimicrobial, and immunomodulating activities at the vaginal level.

Probiotics	Lactoferrin	Polyphenols
Production of bacteriocins Adherence to epithelial cells Generation of mucus components Extracellular matrix-colonization Modulation of the immune system	Increased levels of polydatin-mediated inhibition of lactoferrin in cervicovaginal fluid in FGT infections Reduction of pathogens and inflammatory biomarkers in FGT dysbiosis, following oral vaginal administration	Polydatin-mediated inhibition of NF-kB pathway in murine endometritis Resveratrol-loaded liposomes in vaginal inflammation

## Data Availability

All data for this manuscript have been reported in the paper.

## Abbreviations

AMPs	Antimicrobial peptides
APCs	Antigen-presenting cells
AV	Aerobic vaginitis
BV	Bacterial vaginosis
CSTs	Community state types
DCs	Dendritic cells
D-LA	D-lactic acid
FGT	Female genital tract
HBD	Human beta defensin
HSV	Herpes simplex virus
IL	Interleukin
INOS	Inducible nitric oxide
LF	Lactoferrin
LPS	Lipopolysaccharide
L-LA	L-lactic acid
NK	Natural killer
PID	Pelvic inflammatory disease
ROS	Reactive oxygen species
SCFAs	Short-chain fatty acids
Th	T helper
TGF	Transforming growth factor
TJ	Tight junction
Treg	T regulatory cell
TREM	Tissue resident memory
TSST-1	Toxic shock syndrome toxin-1
